# Gene ontology based transfer learning for protein subcellular localization

**DOI:** 10.1186/1471-2105-12-44

**Published:** 2011-02-02

**Authors:** Suyu Mei, Wang Fei, Shuigeng Zhou

**Affiliations:** 1 Software College, Shenyang Normal University, Shenyang, PR China; 2 Shanghai Key Laboratory of Intelligent Information Processing, School of Computer Science, Fudan University, Shanghai, PR China

## Abstract

**Background:**

Prediction of protein subcellular localization generally involves many complex factors, and using only one or two aspects of data information may not tell the true story. For this reason, some recent predictive models are deliberately designed to integrate multiple heterogeneous data sources for exploiting multi-aspect protein feature information. Gene ontology, hereinafter referred to as *GO*, uses a controlled vocabulary to depict biological molecules or gene products in terms of biological process, molecular function and cellular component. With the rapid expansion of annotated protein sequences, gene ontology has become a general protein feature that can be used to construct predictive models in computational biology. Existing models generally either concatenated the *GO *terms into a flat binary vector or applied majority-vote based ensemble learning for protein subcellular localization, both of which can not estimate the individual discriminative abilities of the three aspects of gene ontology.

**Results:**

In this paper, we propose a Gene Ontology Based Transfer Learning Model (*GO-TLM*) for large-scale protein subcellular localization. The model transfers the signature-based homologous *GO *terms to the target proteins, and further constructs a reliable learning system to reduce the adverse affect of the potential false *GO *terms that are resulted from evolutionary divergence. We derive three *GO *kernels from the three aspects of gene ontology to measure the *GO *similarity of two proteins, and derive two other spectrum kernels to measure the similarity of two protein sequences. We use simple non-parametric cross validation to explicitly weigh the discriminative abilities of the five kernels, such that the time & space computational complexities are greatly reduced when compared to the complicated semi-definite programming and semi-indefinite linear programming. The five kernels are then linearly merged into one single kernel for protein subcellular localization. We evaluate *GO-TLM *performance against three baseline models: *MultiLoc, MultiLoc-GO *and *Euk-mPLoc *on the benchmark datasets the baseline models adopted. 5-fold cross validation experiments show that *GO-TLM *achieves substantial accuracy improvement against the baseline models: 80.38% against model *Euk-mPLoc *67.40% with *12.98% *substantial increase; 96.65% and 96.27% against model *MultiLoc-GO *89.60% and 89.60%, with *7.05% *and *6.67% *accuracy increase on dataset *MultiLoc plant *and dataset *MultiLoc animal*, respectively; 97.14%, 95.90% and 96.85% against model *MultiLoc-GO *83.70%, 90.10% and 85.70%, with accuracy increase *13.44%*, *5.8% *and *11.15% *on dataset *BaCelLoc plant*, dataset *BaCelLoc fungi *and dataset *BaCelLoc animal *respectively. For *BaCelLoc *independent sets, *GO-TLM *achieves 81.25%, 80.45% and 79.46% on dataset *BaCelLoc plant holdout*, dataset *BaCelLoc plant holdout *and dataset *BaCelLoc animal holdout*, respectively, as compared against baseline model *MultiLoc-GO *76%, 60.00% and 73.00%, with accuracy increase *5.25%*, *20.45% *and *6.46%*, respectively.

**Conclusions:**

Since direct homology-based *GO *term transfer may be prone to introducing noise and outliers to the target protein, we design an explicitly weighted kernel learning system (called Gene Ontology Based Transfer Learning Model, *GO-TLM*) to transfer to the target protein the known knowledge about related homologous proteins, which can reduce the risk of outliers and share knowledge between homologous proteins, and thus achieve better predictive performance for protein subcellular localization. Cross validation and independent test experimental results show that the homology-based *GO *term transfer and explicitly weighing the *GO *kernels substantially improve the prediction performance.

## Background

As an important research field in molecular cell biology and proteomics, protein subcellular localization is closely related to protein function, metabolic pathway, signal transduction and biological process, and plays an important role in drug discovery, drug design, basic biological research and biomedicine research. Experimental determination of subcellular localization is time-consuming and laborious, and in some cases, it is hard to determine some subcellular compartments by fluorescent microscopy imaging techniques. Computational methods may help biologist select target proteins and design experiments.

Recent years have witnessed much progress in protein subcellular localization prediction [1-35]. Machine learning methods for predicting protein subcellular localization involve two major aspects: one is to derive protein features and the other is to design predictive model. State-of-art feature extraction methods are data- and model- dependent. We should guarantee that the features not only capture rich biological information but also should be discriminative enough to construct an effective classifier for prediction. On one hand, high throughout sequencing technique makes protein sequences cheaply available, and many computational models are based on protein primary sequences only in computational proteomics. On the other hand, data integration has become a popular method to integrate diverse biological data, including non-sequence information, such as *GO *annotation, protein-protein interaction network, protein structural information, cell image features etc.

There are many effective protein features extracted specifically for protein subcellular localization prediction. Amino acid composition (AA) has close relation with protein subcellular localization [16] and is the most frequently-used features. PseAA [4,10,12,13,17-32] encodes the pair-wise correlation of two amino acids at *λ *intervals using amino acid physiochemical properties. Sliding-window based *k*-mer feature representation is often used to capture the contextual information of amino acid and the conserved motif information, such as gapAA, di-AA, and motif kernel [35,36], etc. Since the dimensionality of *k*-mer feature space (20^*n *^for 20 amino acids) expands exponentially with the window size *n*, some researches [37,38] compress 20 amino acids into 7 groups according to amino acid physiochemical properties. Sorting signal and anchoring signal are important information for protein subcellular localization [39,40], but with the disadvantages that the cleavage sites vary substantially with proteins and the signal peptides may be missing.

Sequence profile is also important information for protein subcellular localization. Marcotte E et al. (2000) [41] revealed the relation between phylogenetic profile distribution and protein subcellular localization pattern. Sequence profile reveals the approximately true amino acid distribution for each amino acid residue along protein sequence, and thus can be naturally used as evolutionary distance between amino acids for measuring the similarity between two protein sequences. Through deliberate design, the similarity between two protein profile distributions can lead to a valid Mercer kernel [14,15,42-46]. Mak M et al. (2008) [42] derived the alignment score between two protein profile distributions using dynamic programming, based on which to derive a valid profile alignment kernel. Profile kernels [43,44] used PSSM & PSFM to derive the similarity score between any two *k*-mers, based on which to measure the similarity between two protein sequences. Kuang R et al. (2005) [44] designed a profile kernel, a variant mismatch kernel [45], which allowed a *k *fragment to match its corresponding *k*-mer if the fragment fell within the positional mutation neighbourhood defined by *k*-mer self-entropy. Kuang R et al. (2009) [46] extended the profile kernel by simple kernel fusion for prediction of malaria degradomes. Spectrum kernel [47] is based on exact *k*-mer match while (*k*, *l*) mismatch kernel [45] allows *l *mismatches within each *k*-mer, both of which are based on protein sequence only without profile incorporation. Actually, we can derive multiple kernels from multi-aspect knowledge about protein and then combine the kernels for more accurate definition of protein similarity. Alexander Z et al. [36] used semi-infinite linear programming to derive the optimal kernel weights for motif kernels combination. Mei S et al. (2010) [48] derived multiple motif kernels from diverse physiochemical constraints on amino acid substitution and combined the kernels for protein subnuclear localization. Kernel method is a good approach for heterogeneous data integration in computational biology.

Although protein sequence contains all the information for proteins to be transported to due compartments, to form correct folding, to form proper 3-D structural conformation and to function properly, etc., quality feature extraction from protein sequence is still a challenging problem because there is no general law or complete knowledge for effective feature extraction from protein sequence. However, large amount of biological experiments and computational inference have accumulated reliable multi-aspect local knowledge about genes and gene products, which has been well organized in the biological knowledgebase: gene ontology (*GO*). Gene ontology is a controlled vocabulary that describes biomolecules and gene products in terms of biological process, function and components. With the rapid progress of experimental and electronic annotation, gene ontology has become a general feature of proteomics that can be used to boost the predictive performance of protein subcellular localization [49-60]. In what follows, we briefly review the *GO*-based predictive models for protein subcellular localization from three viewpoints: (1) from the viewpoint of *GO *term extraction, the previous models can be classified into three categories. The first type of method directly uses protein accession number to query *GO *terms against *GOA *database [61], fast but not applicable to novel proteins [4-12,49-53]. The second type of method uses *PSI-Blast *to transfer the *GO *terms of homologous proteins to the target protein [54,55]. The third method uses *InterProScan *[56] to transfer the *GO *terms of signature proteins to the target proteins [57,58], which may be more reliable than the *PSI-Blast *transfer. Tung T et al. (2009) enlarged the *GO *term coverage by transferring to the target protein the *GO *terms of physically interacting partners in yeast interacting network [59]. (2) From the viewpoint of *GO *feature construction, the previous models also can be classified into three categories. The first way of *GO *feature construction is to simply turn all *GOA GO *terms into a flat binary feature vector to represent proteins [49-53,57-60]. This method has large *GO *term coverage but introduces many *GO *terms irrelevant to the problem concerned. The second type of method uses genetic algorithm to select the most informative *GO *component terms to minimize the irrelevant *GO *terms [54,55], but low *GO *term coverage may be highly likely to turn the test proteins to be null feature vector, so that the effect of *PSI-Blast GO *term transfer would be counteracted. The third type of method does not use explicit *GO *feature representation but designs an implicit kernel function to measure the semantic similarity between two *GO *terms [62]. Actually, the three aspects of gene ontology have different discriminative abilities, but the aforesaid three types of method assume equal feature weight. (3) From the viewpoint of data integration, the previous models can be classified into two categories. The first type of method uses ensemble learning to combine protein sequence with gene ontology, such as *k*-NN ensemble [52], fuzzy *k*-NN [59], and SVM ensemble [62]. The second type of method concatenates all the heterogeneous feature space (e.g. AA, di-AA, gap-AA, chem-AA, *GO*, PPI, etc) into a highly sparse high-dimension feature space [60].

In this paper, we design an explicitly weighted kernel learning system to transfer the known knowledge in terms of *GO *terms from related homology to the target problem, called Gene Ontology Based Transfer Learning Model (*GO-TLM*), for the purpose of sharing knowledge between closely-evolved protein families and achieving better model performance for protein subcellular localization. We use *InterProScan *to conduct multiple homologous signatures based queries against the *InterPro *database, and then transfer the homologous *GO *terms to the target protein. The transferred *GO *terms are potentially prone to errors, partly because of possibly noisy annotations from fluorescent microscopy experiments, electronic annotations using text mining, computational inference, etc. [49], or partly because of the outliers from homology transfer, that is, the homologous proteins actually have distinct function, process and subcellular localization patterns due to evolutionary divergence. Therefore, we should further construct a learning system that is trained on the transferred *GO *terms for reliable prediction. Such a scenario of borrowing knowledge in terms of *GO *terms from homologous proteins for further learning can be viewed as a case of Transfer Learning [63-66], where knowledge is transferred between well-correlated domains for better learning in the target domain. Dai W et al. (2007) [63] proposed an instance-based knowledge transfer learning method, where auxiliary data were drawn in to augment the target training set using *AdaBoost *weighing system to reduce the unfavourable impact of auxiliary data that are subjected to different distribution. Dai W et al. (2008) [64] proposed a feature-based translated transfer learning method, where a translator was constructed between text feature space and image feature space for knowledge transfer from text data to image data. Yang Q et al. (2009) [65] proposed a parameter-based knowledge transfer learning method, where the knowledge contained in annotated image of heterogeneous social web was transferred for target image clustering. Pan S et al. (2010) [66] reviewed the recent progress in transfer learning modelling. Because of the unbalanced knowledge about protein, the three aspects of gene ontology may have distinct discriminative abilities. For this reason, we derive *GO *process features, *GO *function features and *GO *component features individually, and then derive three individual *GO *kernels from the three types of *GO *feature representation. Besides the three *GO *kernels, we further derive another two sequence kernels from amino acid composition (AA) and di-pepetide (di-AA), which are actually spectrum kernel. These heterogeneous feature representations then are then merged into one kernel using linear kernel combination, a classical scenario of multiple kernel learning [36,67]. To reduce the computational cost of parameter optimization for multiple kernel learning, we use simple non-parametric cross validation to estimate the kernel weights instead. The model *GO-TLM *is evaluated against three baseline models on three eukaryotic benchmark datasets using cross validation and independent test.

## Methods

### GO feature construction

The *InterPro *database [68] integrates into a single source the most frequently-accessed signature databases including *PROSITE *[69], *PRINTS *[70], *PFAM *[71], *ProDom *[72], *SMART *[73] and *TIGRFAMs *[74]. *PROSITE *uses regular expression to represent significant amino acid patterns or uses profile (weight matrices) to detect structural and functional domains; *PRINTS *collects protein family fingerprints (motif); *PFAM *is a database of protein domain families that contains curated multiple sequence alignments for each family and corresponding profile hidden Markov models (HMMs); *ProDom *provides automatic domain query that is based on recursive use of *PSI-BLAST *homology search; *SMART *collects domains that are extensively annotated with respect to phyletic distributions, functional class, tertiary structures and functionally important residues; *TIGRFAMs *are a collection of protein families that are characteristic of curated multiple sequence alignments, Hidden Markov Models (HMMs) and associated information supporting functional identification of proteins by sequence homology. *InterProScan *[61] combines different protein signature recognition methods into one resource and provides a uniform web service interface to query signatures against the integrative *InterPro *database. InterProScan provides an option "--goterms" that enables *GO *term query using protein sequence only, which can collect more reliable *GO *terms than *Blast *transfer [54,55]. Parallel access and fast B-tree indexing make *InterProScan *practicable to large problem. For the reason, we use the perl script *InterProScan.pl *as a *GO *term extraction tool. The *GO *term set consists of three subsets: process, function and component. The three *GO *term subsets are organized as three individual binary feature vectors: (*x*_*P*,1_, *x*_*P*,2_,...,*x*_*P*,*l*_); (*x*_*F*,1_, *x*_*F*,2_,...,*x_F,m_*); (*x*_*C*,1_, *x*_*C*,2_,...,*x_C,n_*). It should be noted that *InterProScan *can overcome the problem of data unavailability to a certain degree. If we set high threshold to query more reliable *GO *terms with higher confidence, or the homology also is unannotated, *InterProScan *could neither transfer *GO *terms to the target proteins.

### Kernel weight

*K*-mer occurrence patterns can reveal some conserved sub-sequences (e.g. motif) and *k*-spectrum kernel can be used to define the similarity between protein sequences. Since the feature space expands exponentially with window size |Σ|*^k^*, we only use *1*-mer (AA) and *2*-mer (di-AA) as protein sequence feature representation, thus we can derive kernels *K_AA_*, *K_diAA_*. Based on the *GO *feature representation, we define *GO *process kernel *K_P_*, *GO *function kernel *K_F _*and *GO *component kernel *K_C_*. The 5 kernels are fused into single kernel for more accurate protein similarity definition. Kernel fusion is equivalent to the kernel that is computed in the concatenated feature space, but kernel fusion has the advantage of explicitly weighing the importance of feature subsets. The information content transferred from *GO *kernels to sequence kernels is measured by *GO *kernel weights. The weights of feature subsets vary with problems and should be derived from data. The final kernel is defined as the following linear combination of sub-kernels:

(1) $$K_{GO-TLM}=\sum_{e\in \{P,F,C,AA,diAA\}}w_{e}*K_{e}$$

Lanckriet G et al. (2004) [75] used semi-definite programming to solve the problem, and Alexander Zien et al. (2007) [36] used semi-indefinite linear programming to derive the optimal weights. Both methods have rather large time & space complexity. Here, we use simple non-parametric cross validation to derive the kernel weights *w_e_*,*e *∈ {*P*, *F*, *C*, *AA*, *diAA*}. Given a training data *X*, derive kernels *K_AA_*, *K_diAA_*, *K_P_*, *K_F_*, *K_C _*and split *X *into *K *folds, then conduct *K*-fold cross validation, we can estimate the recall rate or sensitivity (SE) for each kernel. Sensitivity reflects the discriminative ability of kernel or feature subset, but sensitivity is highly biased towards predominant class in the case of unbalanced data, so we should include Matthew's correlation coefficient (MCC) into performance measure to objectively estimate the kernel weights:

(2) $$w_{e}=\frac{SE_{e}*MCC_{e}}{\sum_{c\in \{AA,diAA,P,F,C\}}SE_{c}*MCC_{c}}$$

For denotation simplicity, the subscript *e *is omitted. Assume confusion matrix *M *for some kernel (*K_AA_*, *K_diAA_*, *K_P_*, *K_F_*, *K_C_*), *M_i,j _*records the counts that class *i *is classified to class *j*. Given the following variables that can be derived from the confusion matrix *M*:

(3) $$\begin{array}{l} p_{l}=M_{l,l},q_{l}=\sum_{i=1,i\neq l}^{L}\sum_{j=1,j\neq l}^{L}M_{i,j},r_{l}=\sum_{i=1,i\neq l}^{L}M_{i,l},s_{l}=\sum_{j=1,j\neq l}^{L}M_{l,j},\\ p=\sum_{l=1}^{L}p_{l},q=\sum_{l=1}^{L}q_{l},r=\sum_{l=1}^{L}r_{l},s=\sum_{l=1}^{L}s_{l} \end{array}$$

We can derive the kernel's SE and MCC measure as follows:

(4) $$SE=\frac{\sum_{l=1}^{L}M_{l,l}}{\sum_{i=1}^{L}\sum_{j=1}^{L}M_{i,j}},MCC=\frac{pq-rs}{\sqrt{\left(p+r)(p+s)(q+r)(q+s\right)}}$$

Where, superscript *L *denotes subcellular locations.

As regards with *K_e_*, *e *∈ {*AA*, *diAA*, *P*, *F*, *C*}, Gaussian kernel is used here:

(5) $$K_{e}(x,y)=\exp(\gamma |x-y{|}^{2})$$

*γ *should be fine tuned by experiments.

## Results

### Dataset description

We choose three highly unbalanced eukaryotic benchmark datasets to evaluate *GO-TLM *performance. The first dataset *MultiLoc *collects 5859 proteins that are unevenly distributed to 10 subcellular locations for *Plant *data and 9 subcellular locations for *Fungi *data and *Animal *data [58]; the second dataset *BaCelLoc*, originally from the work [76], collects 491 proteins for *Plant*, 1198 proteins for *Fungi *and 2597 proteins for *Animal *that are unevenly located in 5 subcellular locations for *Plant *and 4 subcellular location for *Fungi *and *Animal *[58,77]; the third dataset *Euk-mPLoc *collects 5618 proteins that are unevenly located in 22 subcellular locations, the largest dataset as far in terms of number of subcellular locations [50]. To overcome overestimation of model performance, a cut-off threshold of 25% sequence similarity is generally accepted in current researches [5-7,13,15,33,34]. In this paper, to allow more training data and as conducted as the baseline models do, 30% threshold of sequence similarity is adopted on all the benchmark datasets, except 40% threshold of sequence similarity for *MultiLoc plant *dataset and 25% threshold of sequence similarity for *Euk-mPloc *dataset.

### Model evaluation and model selection

Among the independent dataset test, sub-sampling (e.g. 5 or 10-fold cross-validation) test and jackknife test (leave-one-out cross validation), the jackknife test is deemed the most objective model evaluation method, as elucidated in [13,15]. Therefore, the jackknife test has been increasingly adopted and widely recognized by investigators to test the power of various prediction methods [1-34]. *5*-fold cross validation is a commonly-accepted model evaluation approach in computational biology for large dataset or complex learning models, whereas leave-one-out cross validation (LOOCV) (i.e. jackknife test) is a better choice for small data or simple computational model. We use 5-fold cross validation to evaluate *GO-TLM *on data *MultiLoc*, *BaCelLoc *and *Euk-mPLoc*, and evaluate *GO-TLM *on *BaCelLoc *independent test as *MultiLoc-GO *did. For 5-fold cross validation, the protein dataset is randomly split into five disjoint parts with equal size. The last part may have 1-4 more examples than the former 4 parts in order for each example to be evaluated on the model. One part of the dataset is used as test set and the remained parts are jointly used as training set. The procedure iterates for five times, and each time a different part is chosen as test set. The independent test is actually hold-out test that randomly partition the dataset into training set and test set. As performance measure, hold-out set is not so objective as cross validation because it does not ensure that each data point is chosen to be tested. For the sake of comparison, we also conduct performance evaluation on *BaCelLoc *independent sets.

As regards to the cross validation for kernel weight evaluation, we select the *cvK *from {3, 5, 10} that achieves best overall accuracy. We use four commonly-adopted measures: Sensitivity (SE), Specificity (SP), Matthew's correlation coefficient (MCC) and Overall Accuracy. MCC is often used to evaluate the performance balance of model prediction. As compared to MCC, Overall Accuracy is a better candidate performance measure for model selection, because it has taken MCC into account. The overall MCC is not given, now that we pay more attention to the bias comparison between sub-categories. LIBSVM (http://www.csie.ntu.edu.tw/~cjlin/libsvm/) is used together with the model *GO-TLM*. The regularization parameter *C *is selected within {2^1^, 2^2^, 2^3^, 2^4^, 2^5^, 2^6^, 2^7^, 2^8^, 2^9^, 2^10^, 2^11^} and the kernel parameter *γ *is selected within {2^-1^, 2^-2^, 2^-3^, 2^-4^}. We adopt the *cvK*, *γ*, *C *combination that achieves the best overall accuracy.

### Comparison with baseline model

We choose *MultiLoc-GO *and *Euk-mPLoc *as baseline models for performance comparison. Both the baseline models incorporated gene ontology information to boost the model's predictive performance. *MultiLoc-GO *used *InterProScan *to draw in *GO *terms while *Euk-mPLoc *used protein accession to directly query *GO *terms against *GOA *database. We use Specificity (SP), Sensitivity (SE), MCC and Overall Accuracy as performance measures.

**Figure 1:**
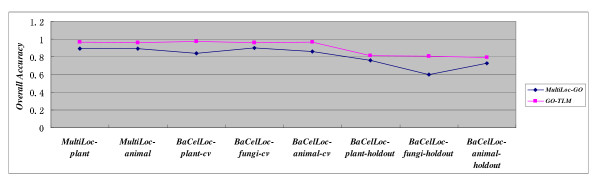
**Performance comparison between *MultiLoc-GO *and *GO-TLM***.

The baseline model *MultiLoc-GO *gave overall accuracy only for cross validation estimation on *MultiLoc *dataset and *BaCelLoc *dataset without detailed SP, SE and MCC. For intuitive illustration of eight comparison experiments between *GO-TLM *and *MultiLoc-GO*, we give performance comparison in a separate chart Figure 1. As can be seen from Figure 1*GO-TLM *significantly outperforms *MultiLoc-GO *on all benchmark datasets. *GO-TLM *achieves quite satisfactory performance for cross validation but significant decrease on *BaCelLoc *independent sets. The accuracy decrease may be caused by the subjective partition of training set and test set. From Figure 1 we can see that *GO-TLM *demonstrates more stable performance than *MultiLoc-GO*. *GO-TLM'*s detailed performance measures see Table 1, 2, 3.

**Table 1:** Performance comparison on 5859 *MultiLoc *protein dataset

*Subcellular location*		*Size*	*MultiLoc*			*MultiLoc-GO*	*GO-TLM*		
			
			SP	SE	MCC	-	SP	SE	MCC
***Fungi*** ***(5407)***	***cytoplasm***	1411	0.68	0.85	0.70	-	0.9181	0.9773	0.9306
	***endoplasmic***	198	0.71	0.59	0.63	-	0.9611	0.8737	0.9136
	***extracellular***	843	0.73	0.81	0.73	-	0.9915	0.9656	0.9749
	***golgi***	150	0.71	0.53	0.60	-	0.9530	0.9467	0.9485
	***mitochondria***	510	0.88	0.82	0.83	-	0.9723	0.9647	0.9655
	***nucleus***	837	0.81	0.74	0.73	-	0.9754	0.9486	0.9558
	***peroxisome***	157	0.68	0.30	0.43	-	0.9735	0.9363	0.9535
	***plasmamembrane***	1238	0.76	0.89	0.78	-	0.9869	0.9774	0.9774
	***vacuole (Astrik)***	63	0.76	0.24	0.42	-	0.9355	0.9206	0.9273

***Plant*** ***(5856)***	***chloroplast (Astrik)***	449	0.88	0.85	0.85	-	1.0000	0.9911	0.9952
	
	***Overall Accuracy***		74.60%			89.60%	**96.65%**		

***Animal*** ***(5547)***	***cytoplasm***	1411	0.67	0.85	0.68	-	0.9221	0.9809	0.9340
	***endoplasmic***	198	0.68	0.56	0.60	-	0.9667	0.8788	0.9189
	***extracellular***	843	0.79	0.83	0.77	-	0.9891	0.9692	0.9753
	***golgi***	150	0.71	0.43	0.53	-	0.9527	0.9400	0.9448
	***mitochondria***	510	0.88	0.82	0.83	-	0.9723	0.9627	0.9642
	***nucleus***	837	0.82	0.73	0.73	-	0.9826	0.9438	0.9566
	***peroxisome***	157	0.71	0.31	0.44	-	0.9799	0.9299	0.9533
	***plasmamembrane***	1238	0.73	0.90	0.76	-	0.9765	0.9750	0.9687
	***lysosome (Astrik)***	103	0.69	0.36	0.48	-	0.9592	0.9126	0.9344
	
	***Overall Accuracy***		74.60%			89.60%	**96.27%**		

**Table 2:** Performance comparison on *BaCelLoc *protein dataset

*Subcellular Location*		*Size*	*Cross validation*				*Independent test*						
			
			*MultiLoc-GO*	*GO-TLM*			*Size* *(train/test)*	*MultiLoc-GO*			*GO-TLM*		
					
			-	SP	SE	MCC		SP	SE	MCC	SP	SE	MCC
***Plant*** ***(491)***	***Nucleus***	121	-	0.9516	0.9752	0.9514	60/61	0.91	0.90	0.77	1.0000	***0.6500***	***0.7252***
	***Cytoplasm***	58	-	0.9434	0.8772	0.8986	52/6	0.41	0.94	0.38	0.7500	1.0000	0.8590
	***Extracellular***	41	-	0.9762	1.0000	0.9869	35/6	0.83	0.95	0.58	1.0000	***0.6667***	0.8100
	***Mitochondria***	67	-	0.9552	0.9552	0.9482	57/10	0.67	0.96	0.51	***0.6429***	***0.9000***	0.7386
	***Chloroplast***	204	-	0.9951	0.9951	0.9916	158/46	0.77	0.94	0.72	***0.7302***	1.0000	0.7777
	
	***Overall Accuracy***		83.70%	**97.14%**			76.00%				**81.25%**		

***Fungi*** ***(1198)***	***Nucleus***	711	-	0.9641	0.9817	0.9354	589/122	0.63	0.79	0.36	1.0000	***0.7213***	0.7246
	***Cytoplasm***	211	-	0.9880	0.9318	0.9564	181/30	0.54	0.78	0.27	***0.483****9*	1.0000	0.6311
	***Extracellular***	88	-	0.9130	0.8957	0.8849	72/16	0.78	0.98	0.60	1.0000	0.9375	0.9653
	***Mitochondria***	188	-	0.9780	0.9570	0.9616	177/11	0.68	0.94	0.62	0.7857	1.0000	0.8786
	
	***Overall Accuracy***		90.10%	**95.90%**			60.00%				**80.45%**		

***Animal*** ***(2597)***	***Nucleus***	1166	-	0.9646	0.9854	0.9551	803/363	0.62	0.93	0.57	0.7965	0.9945	0.7876
	***Cytoplasm***	439	-	0.9402	0.8952	0.9017	302/137	0.72	0.82	0.43	***0.7095***	0.9270	0.7648
	***Extracellular***	804	-	0.9900	0.9900	0.9856	632/172	0.97	0.97	0.89	1.0000	***0.2326***	***0.4406***
	***Mitochondria***	188	-	0.9783	0.9574	0.9653	153/35	0.89	0.97	0.81	0.9714	0.9714	0.9699
	
	***Overall Accuracy***		85.70%	**96.85%**			73.00%				**79.46%**		

**Table 3:** Performance comparison on 5618 *Euk-mPLoc *protein dataset

*Subnuclear location*	*size*	*GO-TLM*		
		
		SP	SE	MCC
***Acrosome***	17	0.9286	0.7647	0.8422
***Cell***	53	0.8085	0.7170	0.7593
***Centriole***	64	0.8958	0.6719	0.7737
***Chloroplast***	501	0.9681	0.9681	0.9650
***Cyanelle***	85	1.0000	0.9882	0.9940
***Cytoplasm***	1060	0.6356	0.7983	0.6475
***Cytoskeleton***	74	***0.2500***	***0.0877***	***0.1431***
***Endoplasmic***	364	0.7453	0.7790	0.7461
***Endosome***	89	0.6591	***0.3867***	***0.4999***
***Extracell***	640	0.7895	0.6402	0.7034
***Golgi***	254	1.0000	0.9231	0.9607
***Hydrogenosome***	13	0.7872	***0.5968***	0.6825
***Lysosome***	80	1.0000	***0.4615***	0.6789
***Melanosome***	13	0.6000	***0.3103***	***0.4295***
***Microsome***	31	0.9349	0.8865	0.9020
***Mitochondrion***	535	0.8071	0.8145	0.7689
***Nucleus***	1333	0.9412	0.8696	0.9032
***Peroxisome***	97	0.8059	0.7781	0.7658
***Plasma***	725	0.9260	0.8410	0.8694
***Spindle pole***	36	0.8750	***0.4118***	***0.5995***
***Synapse***	15	1.0000	***0.5385***	0.7334
***Vacuole***	102	0.9176	0.8571	0.8851

***Overall Accuracy***		**80.38%**		

***Baseline: Euk-mPLoc model***		67.40%		

***Baseline: Fuzzy K-NN model***		62.25%		

On *MultiLoc plant *dataset with 10 subcellular compartments, the best parameter combination is *cvK *= 5, *γ *= 2^-2^, *C *= 2^8 ^and the best Overall Accuracy is 96.55%, 7.05% increase from *MultiLoc-GO *89.60% [58], 22.05% sharp increase from *MultiLoc *74.60% [76]. As can be seen from Table 1*GO-TLM *demonstrates quite satisfactory performances on all the subcellular locations, with SP, SE and MCC all greater than 90%, far better than sequence-based *MultiLoc*. *MultiLoc-GO *gave no detailed cross validation performance measures on each subcellular location. The performance measures *SP*, *SE *and *MCC *demonstrate that *GO-TLM *shows no bias towards large subcellular locations, e.g. the smallest *vacuole *SP: 0.9355, SE: 0.9206, MCC: 0.9273 on *MultiLoc plant*. Similar conclusions can be drawn on *MultiLoc animal*. The best parameter combination is *cvK *= 5, *γ *= 2^-2^, *C *= 2^8 ^for *MultiLoc animal. MultiLoc fungi *dataset shares most proteins with *MultiLoc plant*, without *chloroplast *compartment, so we don't give results on *MultiLoc fungi *dataset.

We conduct two sets of experiments on the second dataset *BaCelLoc*. As can be seen from Table 2 the cross validation experiments show that *GO-TLM *achieves best overall accuracy 97.14%, 95.90% and 96.85% on *BaCelLoc plant*, *BaCelLoc fungi *and *BaCelLoc animal*, respectively against *MultiLoc-GO *83.70%, 90.10% and 85.70%, with accuracy increase 13.44%, 5.8% and 11.15%, respectively. The performance measures *SP*, *SE *and *MCC *demonstrate that *GO-TLM *shows no bias towards large subcellular locations, e.g. the smallest *extracellular *SP: 0.9762, SE: 1.0000, MCC: 0.9869 on *BaCelLoc plant*; the smallest *extracellular *SP: 0.9130, SE: 0.8957, MCC: 0.8849 on *BaCelLoc fungi*; and the smallest *Mitochondria *SP: 0.9783, SE: 0.9574, MCC: 0.9653 on *BaCelLoc animal*. The best parameter combination is *cvK *= 5, *γ *= 2^-2^, *C *= 2^7 ^for *BaCelLoc plant; cvK *= 5, *γ *= 2^-2^, *C *= 2^7^for *BaCelLoc fungi*; and *cvK *= 5, *γ *= 2^-2^, *C *= 2^6 ^for *BaCelLoc animal*. *MultiLoc-GO *gave no detailed SP, SE and MCC performance.

As can be seen in Table 2 the independent test on *BaCelLoc *datasets show that *GO-TLM *achieves 81.25%, 80.45% and 79.46% on *plant*, *fungi *and *animal*, respectively, as compared against *MultiLoc-GO *76%, 60.00% and 73.00%, with accuracy increase 5.25%, 20.45% and 6.46%, respectively. As can be seen from MCC performance, *GO-TLM *generally shows less bias towards large subcellular locations than *MultiLoc-GO*, e.g. *Cytoplasm *(0.8590 vs. 0.38), *Extracellular *(0.8100 vs. 0.58) on *plant*; *Nucleus *(0.7246 vs. 0.36), *Cytoplasm *(0.6311 vs. 0.27) on *fungi*; and *Nucleus *(0.7876 vs. 0.57), *Cytoplasm *(0.7648 vs. 0.43) on *animal*. The improvement on MCC measure may indicate the significance of incorporating MCC measure into *GO-TLM *kernel weight estimation as illustrated in formula (1). At the same time, *GO-TLM *also shows a little performance decrease on several measure values (in bold italic).

On *Euk-mPLoc *data with 22 subcellular compartments, the best parameter combination is *cvK *= 5, *γ *= 2^-3^, *C *= 2^7 ^and the best Overall Accuracy is 80.38%, 12.98% substantial increase from *Euk-mPLoc *67.40% [50] and 18.13% sharp increase from *Fuzzy K-NN *62.25% [59]. *Fuzzy K-NN *was evaluated on the old version of *Euk-mPLoc *with 22 subcellular locations and 4708 proteins. The multi-location proteins are excluded and only its single-location *Measure I *is taken as the comparative baseline here. *Euk-mPLoc *and *Fuzzy K-NN *gave no detailed performance. As can be seen from Table 3*GO-TLM *shows quite satisfactory MCC performance on most subcellular locations, including most small compartments such as *Acrosome *0.8764, *Microsome *0.8923, *Hydrogenosome *0.7747, etc. There are two small compartments that achieve poor MCC performance: *Cytoskeleton *(MCC: 0.1431) &*Melanosome *(MCC: 0.5523). As compared to the previous models, *GO-TLM *can help reduce the bias towards the subcellular locations with larger number of training proteins.

### Kernel weight distribution

**Figure 2:**
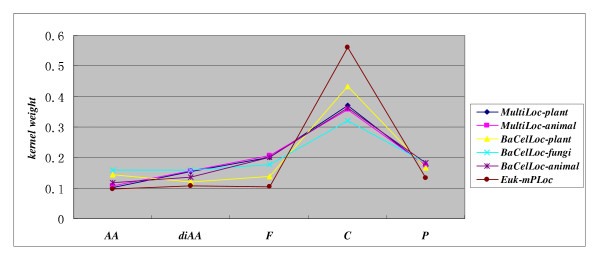
**Kernel weight estimation using 5-fold cross validation**.

**Figure 3:**
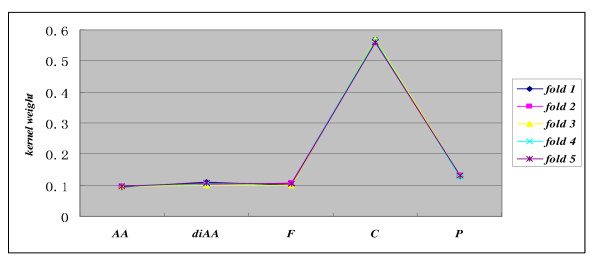
**Kernel weights estimation on *Euk-mPLoc *dataset using 5-fold cross validation**.

The weights for kernel *K_AA_*, *K_diAA_*, *K_P_*, *K_F_*, *K_C _*on the benchmark datasets are illustrated in Figure 2. For each fold of cross validation, the training set is further subjected to *cvK*-fold cross validation to estimate the five kernels' performance measures (SP, SE and MCC), based on which to further estimate the kernels' weights using formula (1). Experiments shows that the kernel weights for 5-fold cross validation vary slightly (take *Euk-mPLoc *dataset for instance, see Figure 3). As can be seen from Figure 2*GO-TLM *demonstrates similar kernel weight distribution on all the benchmark datasets. *GO *features show much stronger discriminative ability than sequence features and the *GO *component terms from signature proteins contribute most to the predictive performance, *GO *process terms the second and *GO *function terms the third. The results may imply that *GO *component terms are more directly indicative of subcellular location than *GO *function terms and *GO *process terms, or the training proteins have less component term missing rate than function and process term missing rate. Take *Euk-mPLoc *dataset for example, there are 658 proteins without *GO *process terms, accounting for 11.71% missing rate; there are 755 proteins without *GO *function terms, accounting for 13.44% missing rate; and there are 31 proteins without *GO *component terms, accounting for 0.56%, far less than the missing rate of function terms and process terms. On the other hand, the weights for *K_AA_*, *K_diAA _*vary little with datasets, while the weights for *K_P_*, *K_F_*, *K_C _*vary widely with datasets, the higher for *K_C _*weight, the lower for *K_P_*, *K_F _*weights. *GO-TLM *achieves the highest *K_C _*weight on *Euk-mPLoc *and the lowest *K_C _*weight on *BaCelLoc-fungi*. The result may also be explained by the missing rate of *GO *terms, e.g. 0 missing rate for *BaCelLoc-fungi *component terms, while 0.56% missing rate for *Euk-mPLoc *component terms. *BaCelLoc-fungi *has less missing rate of process term and function term, and has process weight and function weight slightly increased. We can see that the unbalanced *GO *term distribution contributes much to the variation of *GO *kernel weights.

**Figure 4:**
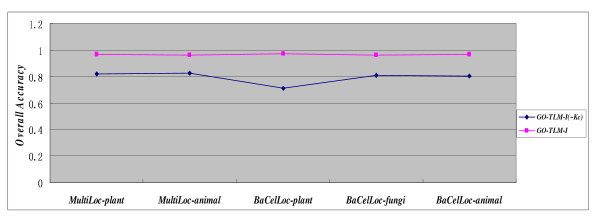
**Performance comparison between *GO-TLM *and *GO-TLM(~Kc)***.

Now that *K_C _*weight is much higher than the other kernel weights, we had better further study the predictive performance of the model that is trained on all the kernels except *K_C _*, referred to as *GO-TLM*(~*K_C_*). The performance comparison between *GO-TLM *and *GO-TLM*(~*K_C_*) is illustrated in Figure 4 which shows that the removal of kernel *K_C _*leads to substantial 14.67%~26.12% performance decrease. The result demonstrates that the *GO *component terms play a critical role in protein subcellular localization. However, the model *GO-TLM-I*(~*K_C_*) achieves over 80% overall accuracy on datasets *MultiLoc-plant, MultiLoc-animal, BaCelLoc-fungi *and *BaCelLoc-animal*, which demonstrates that the other 4 kernels also benefit the protein subcellular localization prediction. Lu Z et al. (2005) has elucidated that GO function terms are good indicator of protein subcellular localization [78].

## Discussion

Traditionally, the knowledge in terms of *GO *terms about homology can be directly transferred to the target proteins based on signature or homology search. Such a way of knowledge transfer generally benefits the research on unknown domain, species or family in biology. However, this process may be prone to introducing noise and outlier, partly because sequence similarity unnecessarily implies similar subcellular localization pattern, molecular function or biological process; or partly because the annotations in themselves may be noisy. For the reason, we design a transfer learning system to share knowledge between homology for reliable protein subcellular localization, called Gene Ontology Based Transfer Learning Model (*GO-TLM*). *GO-TLM *collects *GO *terms based on signature or homology search against the integrative database *InterPro*, and then transfer the *GO *terms to the target proteins for further learning. All the transferred *GO *terms are used to train a kernel-based SVM classifier, which can effectively reduce the risk of outliers by allowing larger training error to achieve maximum margin between two-class separating hyperplanes. Thus, the quite different *GO *terms (e.g. extracell *GO *term is transferred to nuclear proteins) would be viewed as outlier after SVM training. Such a way of constructing a learning system based on the transferred knowledge between related domains or data may benefit computational biology in many aspects. As compared to concatenation of heterogeneous feature subspace, multiple kernel learning has the advantage of explicitly weighing the feature subset/kernel contribution to the classification task. *GO-TLM *uses simple non-parametric cross validation to estimate the kernel weights, serially one kernel in memory at a time, such that it requires much less time and space than the complicated semi-definite/semi-indefinite linear programming. Simple non-parametric cross validation is used to estimate the kernel weights. Meanwhile, the kernel weight estimation allows for both sensitivity and unbalanced measure MCC, such that *GO-TLM *would work better in the scenario of unbalanced training dataset. Experiments reveal that *GO *component feature play more important role than *GO *process feature and *GO *function feature. With less missing rate, *GO *function terms and *GO *process terms would further increase the predictive performance.

*GO-TLM *only uses those *GO *terms that belong to the problem concerned, thus no irrelevant *GO *term is into the *GO *feature vector. However, this method of *GO *feature construction may cause low *GO *term coverage, that's, a test *GO *term (*GO *term that belongs to a test protein) may find no match in the training *GO *term set. In such a scenario, we should include the test *GO *term into the training *GO *term set to re-train the well-trained learning system. Re-training is generally time-consuming for large data and complex model selection. We had better pull in more statistically correlated *GO *terms for those proteins with very few evidences. To avoid re-training, we had better use statistically correlated *GO *term to replace the *GO *term that may not hit the training *GO *terms. Lastly, there is still a large chance for *InterProScan *to miss capturing *GO *terms from homology because of the unevenly distribution of *GO *terms. In such a scenario, we can lower the threshold for *InterProScan *to draw in the *GO *terms from remote homology. Since user-friendly and publicly accessible web-servers represent the future direction for developing practically more useful predictors [79], we shall make efforts in our future work to provide a web-server for the method presented in this paper.

## Conclusions

In this paper, we design an explicitly weighted kernel learning system to transfer the known knowledge in terms of *GO *terms from related homology to the target problem, called Gene Ontology Based Transfer Learning Model (*GO-TLM*), to reduce the risk of outlier and achieve better model performance. On one hand, homology or signature based *GO *term transfer enables reliable knowledge share between homology, protein subfamily or protein family. On the other hand, *GO-TLM *uses simple and effective non-parametric cross validation to explicitly weigh the contribution of the three aspects of gene ontology. The explicitly weighted kernel combination can better cope with the different missing rates and different discriminative abilities between the three aspects of gene ontology. The kernel weight estimation takes into account MCC measure, such that *GO-TLM *could perform better in the scenario of unbalanced data distribution among subcellular locations. Experiments on three benchmark datasets show that *GO-TLM *significantly outperforms the previous models.

## Competing interests

The authors declare that they have no competing interests.

## Authors' contributions

MSY conducted the survey and the computational modelling. WF and SGZ reviewed the study. All authors read and approved the final manuscript.

## References

[B1] Chou KC Elrod DW Protein subcellular location prediction Protein Eng 1999 12 107 118 10.1093/protein/12.2.107 10195282

[B2] Chou KC Cai YD Using functional domain composition and support vector machines for prediction of protein subcellular location J Biol Chem 2002 277 45765 45769 10.1074/jbc.M204161200 12186861

[B3] Xiao X Shao SH Ding YS Huang ZD Chou KC Using cellular automata images and pseudo amino acid composition to predict protein subcellular location Amino Acids 2006 30 49 54 10.1007/s00726-005-0225-6 16044193 PMC7087770

[B4] Shen HB Chou KC A top-down approach to enhance the power of predicting human protein subcellular localization: Hum-mPLoc 2.0 Anal Biochem 2009 394 269 274 10.1016/j.ab.2009.07.046 19651102

[B5] Chou KC Shen HB Hum-PLoc: A novel ensemble classifier for predicting human protein subcellular localization Biochem Biophys Res Commun 2006 347 150 157 10.1016/j.bbrc.2006.06.059 16808903

[B6] Shen HB Chou KC Virus-PLoc: A fusion classifier for predicting the subcellular localization of viral proteins within host and virus-infected cells Biopolymers 2007 85 3 233 240 10.1002/bip.20640 17120237

[B7] Chou KC Shen HB Large-scale plant protein subcellular location prediction J Cell Biochem 2007 100 665 678 10.1002/jcb.21096 16983686

[B8] Chou KC Shen HB Predicting eukaryotic protein subcellular location by fusing optimized evidence-theoretic K-nearest neighbor classifiers Journal of Proteome Research 2006 5 1888 1897 10.1021/pr060167c 16889410

[B9] Shen HB Chou KC Gneg-mPLoc: A top-down strategy to enhance the quality of predicting subcellular localization of Gram-negative bacterial proteins Journal of Theoretical Biology 2010 in press 20093124 10.1016/j.jtbi.2010.01.018

[B10] Chou KC Shen HB Large-scale predictions of gram-negative bacterial protein subcellular locations Journal of Proteome Research 2006 5 3420 3428 10.1021/pr060404b 17137343

[B11] Shen HB Chou KC Gpos-mPLoc: A top-down approach to improve the quality of predicting subcellular localization of Gram-positive bacterial proteins Protein & Peptide Letters 2009 16 1478 1484 10.2174/092986609789839322 20001911

[B12] Chou KC Shen HB Review: Recent progresses in protein subcellular location prediction Anal Biochem 2007 370 1 16 10.1016/j.ab.2007.07.006 17698024

[B13] Chou KC Pseudo amino acid composition and its applications in bioinformatics, proteomics and system biology Current Proteomics 2009 6 4 262 274 10.2174/157016409789973707

[B14] Liu H Yang J Liu DQ Shen HB Chou KC Using a new alignment kernel function to identify secretory proteins Protein & Peptide Letters 2007 14 2 203 208 10.2174/092986607779816087 17305609

[B15] Wang M Yang J Chou KC Using string kernel to predict signal peptide cleavage site based on subsite coupling model Amino Acids 2005 28 395 402 10.1007/s00726-005-0189-6 15838592

[B16] Cedano J Aloy P P'erez-Pons J Querol E Relation between amino acid composition and cellular location of proteins Journal of Molecular Biology 1997 266 594 600 10.1006/jmbi.1996.0804 9067612

[B17] Chou K Prediction of protein subcellular locations by incorporating quasi-sequence-order effect Biochemical and Biophysical Research Communications 2000 278 477 483 10.1006/bbrc.2000.3815 11097861

[B18] Nanni L Lumini A Genetic programming for creating Chou's pseudo amino acid based features for submitochondria localization Amino Acids 2008 34 653 660 10.1007/s00726-007-0018-1 18175047

[B19] Qiu JD Huang JH Liang RP Lu XQ Prediction of G-protein-coupled receptor classes based on the concept of Chou's pseudo amino acid composition: an approach from discrete wavelet transform Analytical Biochemistry 2009 390 1 68 73 10.1016/j.ab.2009.04.009 19364489

[B20] Lin H The modified Mahalanobis discriminant for predicting outer membrane proteins by using Chou's pseudo amino acid composition J Theor Biol 2008 252 350 356 10.1016/j.jtbi.2008.02.004 18355838

[B21] Zeng YH Guo YZ Xiao RQ Yang L Yu LZ Li ML Using the augmented Chou's pseudo amino acid composition for predicting protein submitochondrialocations based on auto covariance approach J Theor Biol 2009 59 366 372 10.1016/j.jtbi.2009.03.028 19341746

[B22] Ding YS Zhang TL Gu Q Zhao PY Chou KC Using maximum entropy model to predict protein secondary structure with single sequence Protein & Peptide Letters 2009 16 552 560 10.2174/092986609788167833 19442235

[B23] Zhou XB Chen C Li ZC Zou XY Using Chou's amphiphilic pseudo-amino acid composition and support vector machine for prediction of enzyme subfamily classes J Theor Biol 2007 248 546 551 10.1016/j.jtbi.2007.06.001 17628605

[B24] Ding YS Zhang TL Using Chou's pseudo amino acid composition to predict subcellular localization of apoptosis proteins: an approach with immune genetic algorithm-based ensemble classifier Pattern Recognition Letters 2008 29 1887 1892 10.1016/j.patrec.2008.06.007

[B25] Chen C Chen L Zou X Cai P Prediction of protein secondary structure content by using the concept of Chou's pseudo amino acid composition and support vector machine Protein & Peptide Letters 2009 16 1 27 31 10.2174/092986609787049420 19149669

[B26] Ding H Luo L Lin H Prediction of cell wall lytic enzymes using Chou's amphiphilic pseudo amino acid composition Protein & Peptide Letters 2009 16 351 355 10.2174/092986609787848045 19356130

[B27] Jiang X Wei R Zhang TL Gu Q Using the concept of Chou's pseudo amino acid composition to predict apoptosis proteins subcellular location: an approach by approximate entropy Protein & Peptide Letters 2008 15 392 396 10.2174/092986608784246443 18473953

[B28] Li FM Li QZ Predicting protein subcellular location using Chou's pseudo amino acid composition and improved hybrid approach Protein & Peptide Letters 2008 15 6 612 616 10.2174/092986608784966930 18680458

[B29] Lin H Ding H Feng-Biao Guo FB Zhang AY Huang J Predicting subcellular localization of mycobacterial proteins by using Chou's pseudo amino acid composition Protein & Peptide Letters 2008 15 No.7 739 744 10.2174/092986608785133681 18782071

[B30] Esmaeili M Mohabatkar H Mohsenzadeh S Using the concept of Chou's pseudo amino acid composition for risk type prediction of human papillomaviruses J Theor Biol 2010 263 2 203 209 10.1016/j.jtbi.2009.11.016 19961864

[B31] Qiu JD Huang JH Shi SP Liang RP Using the concept of Chou's pseudo amino acid composition to predict enzyme family classes: an approach with support vector machine based on discrete wavelet transform Protein & Peptide Letters 2010 17 715 712 10.2174/092986610791190372 19961429

[B32] Gu Q Ding YS Zhang TL Prediction of g-protein-coupled receptor classes in low homology using Chou's pseudo amino acid composition with approximate entropy and hydrophobicity patterns Protein Pept Lett 2010 17 5 559 567 10.2174/092986610791112693 19594431

[B33] Chou KC Shen HB A new method for predicting the subcellular localization of eukaryotic proteins with both single and multiple sites: Euk-mPLoc 2.0 PLoS ONE 2010 5 4 e9931 10.1371/journal.pone.0009931 20368981 PMC2848569

[B34] Chou KC Shen HB Plant-mPLoc: a top-down strategy to augment the power for predicting plant protein subcellular localization PLoS ONE 2010 5 6 e11335 10.1371/journal.pone.0011335 20596258 PMC2893129

[B35] Bhasin M Raghava G ELSpred: SVM-based method for subcellular localization of eukaryotic proteins using dipeptide composition and PSI-BLAST Nucleic Acid Res 2004 32 Web Server W414 W419 10.1093/nar/gkh350 15215421 PMC441488

[B36] Alexander Z Cheng S An automated combination of kernels for predicting protein subcellular localization NIPS 2007, workshop on Machine Learning in Computational Biology

[B37] Dijk A Bosch D Braak C Krol A Ham R Predicting sub-Golgi localization of type II membrane proteins Bioinformatics 2008 24 16 1779 1786 10.1093/bioinformatics/btn309 18562268 PMC7110242

[B38] Shen J Zhang J Luo X Zhu W Yu K Chen K Li Y Jiang H Predicting protein-protein interactions based only on sequences information PNAS 2007 104 11 4337 4341 10.1073/pnas.0607879104 17360525 PMC1838603

[B39] Schneider G Fechner U Review advances in the prediction of protein targeting signals Proteomics 2004 4 1571 1580 10.1002/pmic.200300786 15174127

[B40] Hoglund A Donnes P Blum T Adolph H Kohlbacher O MultiLoc: prediction of protein subcellular localization using N-terminal targeting sequences, sequence motifs and amino acid composition Bioinformatics 2006 22 10 1158 1165 10.1093/bioinformatics/btl002 16428265

[B41] Marcotte E Xenarios I van Der Bliek A Eisenberg D Localizing proteins in the cell from their phylogenetic profiles Proc Natl Acad Sci 1997 12115 12120 10.1073/pnas.220399497 PMC17303 11035803

[B42] Mak M Guo J Kung S PairProSVM: protein subcellular localization based on local pairwise profile alignment and SVM IEEE/ACM Transactions on Computational Biology and Bioinformatics 2008 5 3 416 422 10.1109/TCBB.2007.70256 18670044

[B43] Rangwala H Karypis G Profile-based direct kernels for remote homology detection and fold recognition Bioinformatics 2005 21 23 4239 4247 10.1093/bioinformatics/bti687 16188929

[B44] Kuang R Ie E Wang K Siddiqi M Freund Y Leslie C Profile-based string kernels for remote homology detection and motif extraction J Bioinform Comput Biol 2005 3 527 550 10.1142/S021972000500120X 16108083

[B45] Leslie C Eskin E Cohen A Weston J Noble W Mismatch string kernels for discriminative protein classification Bioinformatics 2004 20 4 467 476 10.1093/bioinformatics/btg431 14990442

[B46] Kuang R Jianying Gu Cai Hong Wang Yufeng Improved prediction of malaria degradomes by supervised learning with SVM and profile kernel Genetica 2009 136 189 209 10.1007/s10709-008-9336-9 19057851 PMC2721224

[B47] Leslie C Eskin E Noble W The spectrum kernel: a string kernel for SVM protein classification Proc Pac Biocomput Symp 2002 7 566 575 11928508

[B48] Mei S Wang Fei Amino acid classification based spectrum kernel fusion for protein subnuclear localization BMC Bioinformatics 2010 11 Suppl 1 S17 10.1186/1471-2105-11-S1-S17 20122188 PMC3009488

[B49] Shen H Yanq J Chou KC Euk-PLoc: an ensemble classifier for large-scale eukaryotic protein subcellular location prediction Amino Acids 2007 33 57 67 10.1007/s00726-006-0478-8 17235453

[B50] Chou KC Shen HB Euk-mPLoc: a fusion classifier for large-scale eukaryotic protein subcellular location prediction by incorporating multiple sites J Proteome Res 2007 6 1728 1734 10.1021/pr060635i 17397210

[B51] Shen HB Chou KC Hum-mPLoc: an ensemble classifier for largescale human protein subcellular location prediction by incorporating samples with multiple sites Biochem Biophys Res Commun 2007 355 1006 1011 10.1016/j.bbrc.2007.02.071 17346678

[B52] Chou KC Shen HB Cell-PLoc: A package of web-servers for predicting subcellular localization of proteins in various organisms Nature Protocols 2008 3 153 162 10.1038/nprot.2007.494 18274516

[B53] Chou K Cai Y A new hybrid approach to predict subcellular localization of proteins by incorporating Gene Ontology Biochem Biophys Res Commun 2003 311 743 747 10.1016/j.bbrc.2003.10.062 14623335

[B54] Huang W Tunq C Ho S Hwang S Ho S ProLoc-GO: utilizing informative gene ontology terms for sequence-based prediction of protein subcellular localization BMC Bioinformatics 2008 9 80 10.1186/1471-2105-9-80 18241343 PMC2262056

[B55] Huang W Tung C Huang H Ho S Predicting protein subnuclear localization using GO-amino-acid composition features BioSystems 2009 19583993 10.1016/j.biosystems.2009.06.007

[B56] Zdobnov EM Apweiler R InterProScan - an integration platform for the signature-recognition methods in InterPro Bioinformatics 2001 17 847 848 10.1093/bioinformatics/17.9.847 11590104

[B57] Chou K Cai Y Prediction of protein subcellular locations by GO-FunD-PseAA predictor Biochem Biophys Res Commun 2004 320 1236 1239 10.1016/j.bbrc.2004.06.073 15249222

[B58] Blum T Briesemeister S Kohlbacher O MultiLoc2: integrating phylogeny and Gene Ontology terms improves subcellular protein localization prediction BMC Bioinformatics 2009 10 274 10.1186/1471-2105-10-274 19723330 PMC2745392

[B59] Tung T Lee D A method to improve protein subcellular localization prediction by integrating various biological data sources BMC Bioinformatics 2009 10 Suppl 1 S43 10.1186/1471-2105-10-S1-S43 19208145 PMC2648781

[B60] Lee K Chuang H Beyer A Sung M Huh W Lee B Ideker T Protein networks markedly improve prediction of subcellular localization in multiple eukaryotic species Nucleic Acids Research 2008 36 20 e136 10.1093/nar/gkn619 18836191 PMC2582614

[B61] Ashburner M Ball CA Blake JA Botstein D Butler H Cherry JM Gene ontology: tool for the unification of biology. The Gene Ontology Consortium Nat Genet 2000 25 25 29 10.1038/75556 10802651 PMC3037419

[B62] Lei Z Dai Y Assessing protein similarity with Gene Ontology and its use in subnuclear localization prediction BMC Bioinformatics 2006 7 491 10.1186/1471-2105-7-491 17090318 PMC1660555

[B63] Dai W Yang Q Xue G Yu Y Boosting for Transfer Learning Proceedings of the 24 th International Conference on Machine Learning 2007

[B64] Dai W Chen Y Xue G Yang Q Yu Y Translated Learning: Transfer Learning across Different Feature Spaces NIPS 2008

[B65] Yang Q Chen Y Xue G Dai W Yu Y Heterogeneous Transfer Learning for Image Clustering via the Social Web Proceedings of the 47th Annual Meeting of the ACL and the 4th IJCNLP of the AFNLP 2009 1 9

[B66] Pan S Yang Q A Survey on Transfer Learning IEEE Transactions on Knowledge and Data Engineering 2010 22 10 1345 1359 10.1109/TKDE.2009.191

[B67] Alexander Z Cheng S Multiclass Multiple Kernel Learning Proceedings of the 24th International Conference on Machine Learning

[B68] Apweiler R Attwood T Bairoch A Bateman A Birney E Biswas M The InterPro database, an integrated documentation resource for protein families, domains and functional sites Nucleic Acids Research 2001 29 1 37 40 10.1093/nar/29.1.37 11125043 PMC29841

[B69] Hofmann K Bucher P Falquet L Bairoch A The Prosite Database, Its Status in 1999 Nucleic Acids Res 1999 27 1 215 219 10.1093/nar/27.1.215 9847184 PMC148139

[B70] Attwood TK Croning MD Flower DR Lewis AP Mabey JE Scordis P The Database Formerly Known as Prints Nucleic Acids Res 2000 28 1 225 227 10.1093/nar/28.1.225 10592232 PMC102408

[B71] Bateman A Birney E Durbin R Eddy SR Howe KL Sonnhammer EL The Pfam Protein Families Database Nucleic Acids Res 2000 28 1 263 266 10.1093/nar/28.1.263 10592242 PMC102420

[B72] Corpet F Gouzy J Kahn D Recent Improvements of the Prodom Database of Protein Domain Families Nucleic Acids Res 1999 27 1 263 267 10.1093/nar/27.1.263 9847197 PMC148152

[B73] Schultz J Copley RR Doerks T Ponting CP Bork P A Web-Based Tool for the Study of enetically Mobile Domains Nucleic Acids Res 2000 28 1 231 234 10.1093/nar/28.1.231 10592234 PMC102444

[B74] Haft DH Loftus BJ Richardson DL Yang F Eisen JA Paulsen IT White O TIGRFAMs: a protein family resource for the functional identification of proteins Nucleic Acids Res 2001 29 1 41 3 10.1093/nar/29.1.41 11125044 PMC29844

[B75] Lanckriet G DeBie T Cristianini N Jordan M Noble W A statistical framework for genomic data fusion Bioinformatics 2004 20 16 2626 2635 10.1093/bioinformatics/bth294 15130933

[B76] Hoglund A Donnes P Blum T Adolph H Kohlbacher O MultiLoc: prediction of protein subcellular localization using N-terminal targeting sequences, sequence motifs and amino acid composition Bioinformatics 2006 22 10 1158 1165 10.1093/bioinformatics/btl002 16428265

[B77] Pierleoni A Luigi P Fariselli P Casadio R BaCelLo: a balanced subcellular localization predictor Bioinformatics 2006 22 14 e408 e416 10.1093/bioinformatics/btl222 16873501

[B78] Lu Z Hunter L GO molecular function terms are predictive of subcellular localization Pac Symp Biocomput 2005 151 61 full_text 15759622 10.1142/9789812702456_0015 PMC2652875

[B79] Chou KC Shen HB Review: recent advances in developing web-servers for predicting protein attributes Natural Science 2009 2 63 92 http://www.scirp.org/journal/NS/(openly accessible at) 10.4236/ns.2009.12011

